# Lower-extremity resistance training on unstable surfaces improves proxies of muscle strength, power and balance in healthy older adults: a randomised control trial

**DOI:** 10.1186/s12877-016-0366-3

**Published:** 2016-11-24

**Authors:** Nils Eckardt

**Affiliations:** Department of Training and Movement Science, Institute of Sport and Sports Science, University of Kassel, Damaschkestraße 25, 34121 Kassel, Germany

**Keywords:** Instability resistance training, Machine-based resistance training, Free-weight resistance training, Unstable surfaces, Seniors

## Abstract

**Background:**

It is well documented that both balance and resistance training have the potential to mitigate intrinsic fall risk factors in older adults. However, knowledge about the effects of simultaneously executed balance and resistance training (i.e., resistance training conducted on unstable surfaces [URT]) on lower-extremity muscle strength, power and balance in older adults is insufficient. The objective of the present study was to compare the effects of machine-based stable resistance training (M-SRT) and two types of URT, i.e., machine-based (M-URT) and free-weight URT (F-URT), on measures of lower-extremity muscle strength, power and balance in older adults.

**Methods:**

Seventy-five healthy community-dwelling older adults aged 65–80 years, were assigned to three intervention groups: M-SRT, M-URT and F-URT. Over a period of ten weeks, all participants exercised two times per week with each session lasting ~60 min. Tests included assessment of leg muscle strength (e.g., maximal isometric leg extension strength), power (e.g., chair rise test) and balance (e.g., functional reach test), carried out before and after the training period. Furthermore, maximal training load of the squat-movement was assessed during the last training week.

**Results:**

Maximal training load of the squat-movement was significantly lower in F-URT in comparison to M-SRT and M-URT. However, lower-extremity resistance training conducted on even and uneven surfaces meaningfully improved proxies of strength, power and balance in all groups. M-URT produced the greatest improvements in leg extension strength and F-URT in the chair rise test and functional reach test.

**Conclusion:**

Aside from two interaction effects, overall improvements in measures of lower-extremity muscle strength, power and balance were similar across training groups. Importantly, F-URT produced similar results with considerably lower training load as compared to M-SRT and M-URT. Concluding, F-URT seems an effective and safe alternative training program to mitigate intrinsic fall risk factors in older adults.

**Trial registration:**

This trial has been registered with clinicaltrials.gov (NCT02555033) on 09/18/2015.

**Electronic supplementary material:**

The online version of this article (doi:10.1186/s12877-016-0366-3) contains supplementary material, which is available to authorized users.

## Background

In the course of ageing, physical abilities decline [1] and consequently there is an increase in risk of falling and fall incidences [1, 2]. Notwithstanding the fact that causes of falls are multifactorial, losses in lower-extremity muscle strength, power and balance seem to be the most prominent intrinsic (i.e., person-related) fall risk factors in older adults [1, 2]. Several systematic reviews and meta-analyses [3–5] highlighted the positive effects of resistance and balance training when applied as a single means, on measures of leg muscle strength, power and balance in older adults. Balance training for example, positively affects static/dynamic steady-state and proactive balance in older adults [6]. Likewise, resistance training has positive effects on measures of muscle strength [7] and balance [8] in older adults. Combinations of resistance and balance training describe in general a consecutive order, where resistance and balance exercises are executed within the same training session or within the same training block. Those exercise interventions have also shown positive effects on measures of strength, power and balance in older adults [9–11].

Besides resistance training and balance training applied as a single means and the combination thereof, resistance training conducted on unstable surfaces (URT) poses an alternative or complimentary means to improve measures of strength, power and balance. URT combines unstable devices (e.g., Swiss balls, BOSU^®^ balls, wobble boards, etc.) and an external load (e.g., body weight, barbell, dumbbell) within one exercise (e.g., squats on a foam block). Because of the instability-related reduction of force, power production and movement velocity [12, 13] during URT when compared to traditional resistance training on stable surfaces (SRT), it was previously argued that URT lacks sufficient strain to induce adaptive stimuli [14]. Several studies however, investigating muscular activity during the performance of strength exercises on stable and unstable surfaces demonstrated similar or even higher muscle activation in URT as compared to SRT [13, 15]. According to Behm and Colado [16], there are two components to URT: balance and load/resistance. The balance component of URT seems to activate stabilising muscles of the core and trigger stabilising function of prime movers in response to greater postural challenges [16, 17]. In consequence, URT can generate appropriate stress to exceed training thresholds and ensure neuromuscular adaptive processes. For example, Kibele and Behm [11] found superior improvements in the single leg hop test following URT compared to SRT in healthy young adults. In line with the principle of training specificity [18] they concluded that URT induced higher additional balance and stabilising adaptations, which were more prominent in the balance demanding single leg hop test. Yet the feasibility and effectiveness of URT compared to SRT on measures of lower-extremity strength, power and balance is insufficient in older adults.

Studies that examined the effects of URT in older adults found meaningful improvements in measures of strength, power, and balance [19–21]. However, two of these studies [19, 21] focused on strengthening the core and improving mainly balance abilities. The other [20] used an unstable device to strengthen lower-extremities. None of these studies compared the effects of URT to traditional SRT, but to non-exercising control groups. A recent review by Behm and colleagues [14] stated that studies comparing URT and SRT were found for young adults but not for older adults. A further notable point is that the aforementioned studies did not use additional loads within their training program. For example Granacher and colleagues [19] examined the effects of a 9-week core instability strength training programme on measures of trunk muscle strength, spinal mobility, dynamic balance, and functional mobility in older adults (63–80 years). They found significant improvements in measures of strength, dynamic balance, and functional mobility. This was in favour of the training group as compared to the non-exercising control group. Another study investigated the effects of a 12-week Swiss ball exercise programme in older adults (≥65 years) and detected positive effects on measures of physical fitness and balance in comparison to a non-exercising control group [21]. Using a slightly different approach, Chulvi-Medrano and colleagues [20] applied an 8-week lower-limb strength training programme in healthy elderly women (>65 years) using an unstable T-Bow^®^ device. The training group showed significant improvements in measures of dynamic, static, and overall balance, whereas the non-exercising control group experienced a decline or no change. Although there are studies available that are engaged with URT in older adults, the lack of comparison to established SRT and the lack of additional load necessitates further investigation.

This study therefore examined the effects of two types of URT in comparison to traditional machine-based SRT on measures of lower-extremity strength, power and balance in older adults. The URT groups were subclassified into machine-based resistance training on stable surfaces (M-URT) and free-weight resistance training on unstable surfaces (F-URT) to explore how different degrees of instability trigger adaptive responses of the neuromuscular system in measures of lower-extremity muscle strength, power and balance. Based on previous studies [14] URT is assumed to have an extra effect on measures of lower-extremity muscle strength, power and balance in older adults.

## Methods

### Participants

**Fig. 1:**
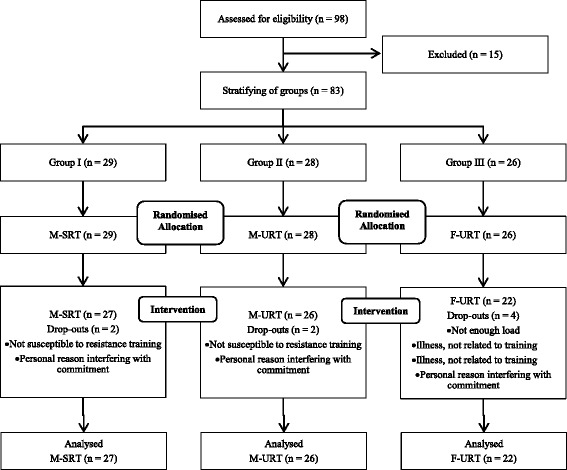
Flow chart of the study protocol according to the CONSORT statements

Eighty-three (48 female, 35 male) community-dwelling older adults aged 65 to 80 years were included in a stratified-randomised control trial [22]. Recruitment was carried out by placing an advertisement in the local newspaper and during a public information meeting at the local town hall. Eligibility was tested according to the recommendations of Gschwind and colleagues [23]. Inclusion criteria were determined as the ability to walk independently without any gait aid. To safeguard participants and account for possible cognitive and mental health conditions or any other neurological, musculoskeletal or heart-related disease, participants were excluded based on pathological ratings of the Clock Drawing Test (CDT), the Mini-Mental-State-Examination (MMSE, < 24 points), the Falls Efficacy Scale – International (FES-I, > 24 points), the Geriatric Depression Scale (GDS, > 9 points), the Freiburg Questionnaire of Physical Activity (FQoPA, < 1 h) and the Frontal Assessment Battery (FAB-D, < 18 points). Participants’ baseline characteristics are presented in Table 1. Figure 1 shows a flow chart of the study design. The study was approved by the local ethics committee of the University of Kassel (E05201401) and was carried out in accordance with ethical standards of the latest Declaration of Helsinki (WMA, Oct. 2013). Prior to the commencement of the study, written informed consent was obtained after providing information on aims and potential risks of the investigation. The study was designed according to the CONSORT publishing and reporting guidelines [24].

**Table 1:** Characteristics of participants at baseline

Characteristic	M-SRT (*n* = 27)		M-URT (*n* = 26)		F-URT (*n* = 22)		Baseline difference
	M	SD	M	SD	M	SD	*p*-value
Age (years)	71.3	4.1	70.0	4.4	69.8	4.3	.409
Body height (cm)	170.1	8.3	172.9	8.6	169.8	7.4	.408
Body mass (kg)	76.7	15.7	77.7	13.1	74.7	13.0	.756
Body mass index (kg/m^2^)	26.9	3.7	25.9	3.4	25.8	3.8	.952
Sex (f/m)	17/10		13 / 13		13 / 9		
Physical activity (h/week)	9.3	4.7	10.5	8.4	9.8	5.0	.820
MMSE	28.4	1.3	28.5	1.1	27.9	1.4	.275
CDT	all participants were classified as non-pathological						
GDS	1.2	0.2	0.7	0.2	1.1	0.2	.127
FAB_D	16.0	1.8	16.2	1.9	16.6	1.4	.550

*M-SRT* machine based resistance training on stable surfaces; *M-URT* machine based resistance training on unstable surfaces; *F-URT* free-weight resistance training on unstable surfaces; *M* mean; *SD* standard deviation; *f* female; *m* male; *MMSE* Mini Mental State Examination; *CDT* Clock Drawing Test; *GDS* Geriatric Depression Scale; *FAB_D* Frontal Assessment Battery, German Version

### Measures

Data was collected in the biomechanics laboratory of the University of Kassel, Germany. Strength, power and balance assessments were conducted by NE and a student assistant. Questionnaires were completed in separated rooms by different student assistants. The allocation of participants to the assessors was carried out randomly. All tests were explained and conducted using standardised verbal instructions regarding the test procedure. All assessors, except NE, were unaware of a participant’s group affiliation during post-testing. A single assessment lasted 90 min per participant.

Maximal isometric leg extension strength, the functional reach test and the chair rise test were considered to be primary measures, as they are thoroughly investigated and therefore reliable measures.

### Assessment of strength and power performance

Well established clinical and biomechanical tests were administered to measure outcomes in muscle strength and power. In accordance with the recommendations of Granacher et al. [19], strength and power assessments were performed after balance assessments to reduce interfering effects of muscle fatigue. Strength, power and balance tests were administered in a randomised order within each respective block. One practice trial was provided for every test. Test procedures were conducted according to the recommendations of Gschwind et al. [23] if not stated otherwise. Two test trials were carried out using the mean for further statistical analysis, except for maximal isometric leg extension strength (ILES) and hand grip strength. In this case the better value of two consecutive trials was used for statistical analysis. Sufficient (at least one minute) recovery periods were provided between trials to limit the effects of fatigue.

Maximal isometric leg extension strength (ILES) was examined with a cable pull device (Takei A5002, Fitness Monitors, Wrexham, England) in an upright body posture. To ensure upright posture participants were instructed to maintain contact between their shoulder and the wall and to resist lifting their scapula while pulling. Individual cable lengths were chosen to ensure a knee angle of approximately 135°. Participants were asked to “pull initially with a moderate intensity and slowly increase the intensity to maximum exertion while keeping the upper body extended and upright”. The test was repeated with at least one minute intervals between measurements. The ILES shows excellent test-retest reliability (ICC = .98) for leg extension strength [25].

To measure handgrip strength a Takei hand dynamometer (Takei A5401, Fitness Monitors, Wrexham, England) was used. Participants stood upright with their arm aligned to the body and squeezed the device as hard as they could, using their dominant hand. The width of the handle was adjusted to the participant’s hand size. In order to do this, the intermediate phalanges had to be placed on the inner handle. The Takei handy dynamometer maintains excellent test-retest reliability (ICC = .95) [26].

In addition to isometric strength assessment, power tests were conducted. Supplementary to the standard Chair Rise Test (CRT) on stable surfaces, test trials were also recorded while standing on a foam pad (AIREX^©^). Participants were instructed to stand up and sit down five times as quickly as possible without using their arms. They were advised to fold their arms across the upper body. Time was measured by a stopwatch to the nearest .01 s. After the countdown “ready-set-go”, testing time was started and stopped when participants were sitting down for the fifth time. For the CRT, high test-retest reliability has been shown (ICC = .89) [27].

In addition, a stair climb power test (SCPT) was administered. This test has shown meaningful associations with mobility performance and strength measures in older adults [28]. Participants were instructed to walk quickly but safely up and down a nine step flight of stairs (step height: 17 cm). Time was started after the cue to go and stopped when the second foot reached the top step and/or the floor, respectively. Use of the handrail was permitted for safety reasons. Time was measured with an ordinary stopwatch to the nearest .01 s. Ascent and descent times were recorded separately and power was calculated with the following formula: $\mathrm{P}=\frac{\left(\mathrm{M}\ \mathrm{x}\ \mathrm{D}\right) \times \mathrm{g}\ }{\mathrm{t}}$, where P = power (Watt), M = body mass (kg), D = vertical distance covered (m), t = time (s) and g = 9.8 m/s^2^ (constant of gravity). Test-retest reliability is shown to be excellent (ICC = .99) [28].

### Assessment of balance performance

Proactive balance was tested using the timed-up-and-go test (TUG) and the functional reach test (FRT). For the TUG, participants were asked to rise from a chair and walk three meters at their normal walking speed around a cone, return to the chair and sit down. Time for the TUG was recorded with a stopwatch to the nearest .01 s on the command “ready-set-go” and stopped as soon as the participants sat down. The TUG has shown excellent test-retest reliability (ICC = .99) in older adults [29]. The FRT measures the maximal distance participants were able to reach forward while standing. For this test participants were instructed to lift their dominant arm and to reach forward as far as they could without taking a step forward. Maximal reach distance (cm) was recorded. The FRT showed excellent test-retest reliability with older adults (ICC = .92) [30]. In addition to the standard FRT on stable surfaces, test trials were recorded while standing on a foam pad (AIREX^©^).

Dynamic steady-state balance was tested while walking on a 10-m walkway, measuring temporal-spatial gait variables, i.e., stride length (cm), stride velocity (m/s), stride width (cm), and double support (%), using a two-dimensional OptoGait^©^ system (Microgate, Bolzano, Italy). In addition, the coefficient of variation (CV) was calculated as follows: CV (%) = (SD/mean) × 100. The gait variables showed high concurrent validity between the OptoGait^©^ system and a previously validated system (ICCs = .93 to .99) [31]. Participants were asked to walk for 10 m in their own footwear at a self-selected pace three times to calculate test-retest reliability. A three-minute interval was given between individual trials to rest, save the data as well as to prepare for the next trial. At the start and the end of the walkway, sufficient distance was provided to accelerate and decelerate safely. In addition, the first and last step was excluded from the analysis to eliminate possible acceleration and deceleration bias. Each trial was recorded at 1000 Hz using the manufacture approved OptoGait^©^ software, running on a laptop computer (Lenovo ThinkPad T530).

To test reactive balance the Push and Release Test (PRT) was used. The PRT rates the postural response to a sudden perturbation. Participants were instructed to push backwards against the examiner’s hands and to regain their balance after the examiner releases his hands. The number of steps required to regain balance was counted and the corresponding score was recorded (0 = 1 step, 1 = 2–3 small steps backwards with independent recovery, 2 = ≥4 steps with independent recovery, 3 = steps with assistance for recovery, 4 = fall or unable to stand without assistance). The PRT has shown high test-retest reliability (ICC = .84) [32].

### Cognitive measures

Psychosocial functions were assessed using several questionnaires. Global cognition was tested using the 30-point MMSE, which is a test for assessing cognitive function and has shown high test-retest reliability (ICC = .89) [33]. The MMSE tests seven cognitive domains: orientation, registration, attention and calculation, recall, language and simple command following [33]. The CDT and FAB-D [34, 35] were used to assess executive function. The CDT is a screening tool for cognitive impairment and a measure of spatial dysfunction. Participants are asked to draw a clock with all its components and a self-selected time. Inter-rater reliability of the CDT has been shown to be excellent (IRR = .92) with sensitivity and specificity values of 50 and 84%, respectively [36]. The FAB-D consists of six neuropsychological tasks, evaluating executive function. Excellent inter-rater reliability has been found for the FAB-D too (IRR = .87). Furthermore, internal consistency has been found to be good (*r* = .78).

### Questionnaires

Fall self-efficacy was measured using the German version of the FES-I [37, 38]. This test has shown excellent internal validity (Cronbach’s alpha = .96) and test-retest reliability (ICC = .96) when assessing the level of fear of falling [37, 39]. To assess health-related physical activity, exercise and the amount of energy expenditure, FQoPA was conducted [40]. Frey and colleagues [41] have demonstrated that FQoPA score correlates with maximum oxygen uptake, indicating high validity (*r* = .42).

### Exercise interventions

Participants were stratified into three intervention groups based on equal distribution of age and gender ratio and baseline values. The allocation to one of the three training programmes occurred randomly, using a random generator [42]. Intervention group one followed a ‘traditional’ machine-based stable resistance training programme (M-SRT). Intervention group two (machine-based unstable resistance [M-URT]) followed a similar training programme with exercise-machines, but with additional unstable devices placed between the participant and the exercise-machine or floor respectively. The third intervention group conducted free-weight resistance training on unstable devices (F-URT) using dumbbells instead of exercise-machines. According to Behm and colleagues [12, 43, 44], free-weight resistance training inherits a certain degree of instability in comparison to machine-based resistance training. In consequence implementing different degrees of instability is achieved by distinguishing machine-based resistance training using unstable devices (i.e., a moderate degree of instability) and free-weight resistance training using unstable devices (i.e., a high degree of instability).

All intervention groups trained for 10 weeks, twice per week on non-consecutive days for 60 min each. The 10-week intervention period consisted of a 1 week introductory phase and three major training blocks lasting 3 weeks each. Training intensity was progressively and individually increased over the 10-week training programme by modulating load and sets for all groups and level of instability for groups M-URT and F-URT. After week one, four, and seven the training load (weight) was increased following one repetition maximum (1-RM) testing for each major exercise. Since the load of a 1-RM is too heavy for untrained elderly, training load was calculated using the prediction equation provided by Epley (as cited in Reynolders, Gordon, Robergs, [45]), showing .03% deviation of actual achieved 1-RM in squats with a correlation of *r* = .97 [46]. Instructors ensured that repetitions did not exceed 15–20, because 1-RM predication accuracy is higher with fewer repetitions [46]. Training under unstable surface conditions, especially with additional weight, implies a certain degree of accident risk. Due to this factor all instability exercises were observed by instructors and made secure with additional aids like boxes. Training was supervised by skilled instructors at all times. For the first two weeks the participant to instructor ratio was 5:1, thereafter 10:1.

Since effectiveness of resistance training when applied as a single means, in comparison to a non-exercising control group on measures of lower-extremity strength, power and balance has been frequently reported in randomised controlled studies [47, 48], reviews [49–51], and meta-analyses [3, 52, 53] M-SRT served as an active control group.

All three intervention groups conducted a resistance training programme consisting of three main exercises, a preparation and cool-down phase. Participants performed ten minutes low-intensity stepping on a stair-walker as a brief warm-up at the beginning of each training session. The main part of the intervention exercises focused on strengthening lower-extremity muscles. Suitably squat-movements were chosen, as recommended by Flanagan and colleagues [54]. M-SRT and M-URT groups performed squats on a Smith machine, fixing the barbell at hip level. Pilot testing revealed that shoulder and lower back mobility of elderly was too limited for resting the barbell on the shoulders. In addition, the M-URT group used instability devices (e.g., BOSU balls, wobble boards, inflatable discs) placed under their feet. Instability devices were also used in the F-URT group, but they performed the squat with dumbbells instead of a barbell. As a secondary lower-extremity exercise, leg-press for the M-SRT and M-URT (using instability devices placed between the feet and foot plate) was chosen. The front lunge (with dumbbells) was conducted as a secondary exercise by the F-URT group. To strengthen the trunk, the bridge exercise was incorporated into the training programme. Again, group M-URT and F-URT additionally used instability devices placed under the shoulders and feet. A detailed description of the training programme, machines, and equipment is outlined in Table 2.

**Table 2:** Intervention programmes

	Intro-phase (1 week)	Block I (3 weeks)	Block II (3 weeks)	Block III (3 weeks)
	~1–2 × 10 reps (with low weights)	2 × 12 reps (50% of the 1-RM)	3 × 12 reps (60% of the 1-RM)	4 × 12 reps (60% of the 1-RM)
M-SRT				
Stair walker	10 min	10 min	10 min	10 min
Smith machine	<150°	<120°	<120°	<120°
Leg-press	90°	90°	90°	90°
Bridge exercise	–	–	–	–
Walking with dumbbells 5 × 10 m	–	10% of bw	15% of bw	20% of bw
Ergometer (2 × 2 min)	1.00 w/kg bw	1.25 w/kg bw	1.50 w/kg bw	1.75 w/kg bw
M-URT				
Stair walker	10 min	10 min	10 min	10 min
Smith machine	<150° + AIREX^®^ coordination rocker board round	<120° + AIREX^®^ coordination rocker board angled	<120° + wooden rocker	<120° + BOSU^®^ ball
Leg-press	90° + THERABAND^™^ stability trainer green	90° + THERABAND^™^ stability trainer green or TOGU^®^ balance block	90° + TOGU^®^ balance block TOGU^®^ DYNAIR^®^	90° + small single wooden rocker
Bridge exercise	–	TOGU^®^ DYNAIR^®^ (under feet)	+ TOGU^®^ DYNAIR^®^ (under shoulder) & BOSU^®^ (under feet)	+ swiss ball (under feet)
Walking with dumbbells 5 × 10 m	on terrasensa^®^ flats	on terrasensa^®^ flats with 10% of bw	on terrasensa^®^ classics with 15% of bw	on terrasensa^®^ classics with 20% of bw
Ergometer (2 × 2 min)	1.00 w/kg bw	1.25 w/kg bw + TOGU^®^ DYNAIR^®^	1.50 w/kg bw + TOGU^®^ DYNAIR^®^	1.75 w/kg bw + TOGU^®^ DYNAIR^®^
F-URT				
Stair walker	10 min	10 min	10 min	10 min
Squats	AIREX^®^ coordination rocker board round	AIREX^®^ coordination rocker board angled	wooden rocker	BoSu ball
Front lunges	AIREX^®^ coordination rocker board round (front foot)	AIREX^®^ coordination rocker board angled (front foot)	TOGU^®^ balance block (front foot) & AIREX^®^ balance pad (rear foot)	TOGU^®^ balance block (front foot) & AIREX^®^ balance pad (rear foot)
Bridge exercise	–	TOGU^®^ DYNAIR^®^ (under feet)	+ TOGU^®^ DYNAIR^®^ (under shoulder) & BOSU^®^ (under feet)	+ swiss ball (under feet)
Walking with dumbbells 5 × 10 m	on terrasensa^®^ flats	on terrasensa^®^ flats with 10% of bw	on terrasensa^®^ classics with 15% of bw	on terrasensa^®^ classics with 20% of bw
Ergometer (2 × 2 min)	1.00 w/kg bw	1.25 w/kg bw + TOGU^®^ DYNAIR^®^	1.50 w/kg bw + TOGU^®^ DYNAIR^®^	1.75 w/kg bw + TOGU^®^ DYNAIR^®^

*M-SRT* machine based resistance training on stable surfaces; *M-URT* machine based resistance training on unstable surfaces; *F-URT* free-weight resistance training on unstable surfaces; *bw* body weight; *w/kg bw* 1 W per kg body weight; *1-RM* one repetition maximum; *BOSU* BOth Sides Utilized

### Statistics

An a priori power analysis using G*Power 3.1 [55] with an assumed type I error of .05 and a type II error of .10 (90% statistical power, correlation among groups: .5, nonsphericity correction: 1) was computed to determine an appropriate sample size to detect medium (.50 ≤ *d* ≤ .79) interaction effects. The calculations were based on a study assessing the effects of core strength training using unstable devices in older adults [19]. The analysis revealed the requirement of 54 participants (18 per group) to obtain medium “time × group” interaction effects. Considering the likelihood of dropouts, 83 participants were recruited to compensate for a possible dropout rate of ~20%.

Prior to the main analysis, normal distribution was checked by visual inspection and tested with the Shapiro Wilk test for each dependent variable. In addition, Levene’s test for equality of variance was conducted. Baseline differences were tested between groups with a one-way ANOVA or a Kruskal-Wallis test depending on distribution and homogeneity. No differences were found (*p* ≥ .067). A 3 (group: M-SRT, M-URT, F-URT) × 2 (time: pre-test, post-test) ANOVA with repeated measures on time was conducted. Ryan-Holm-Bonferroni [56] adjusted post-hoc tests (dependent *t*-tests, Wilcoxon tests) were used to detect statistically significant pre- to post-test differences within each group. Ryan-Holm-Bonferroni corrected *p*-values were reported. In the case of distribution or homogeneity violations, Kruskal-Wallis one-way analyses of variance and Friedman tests were performed for non-parametrical variables and to control results of parametrical tests. If differences were detected, non-parametric test results would be expressed. In addition, differences in absolute training intensity within the last training block were analysed. Therefore, the training load of the squat movement, which was a common exercise to all groups, was used. Differences were computed using a one-way ANOVA. Post-hoc applied independent *t*-tests were used to identify differences between groups. Changes for all variables within groups were calculated with the formula ∆% = ((Mean_pre_/Mean_post_) – 1) × 100.

To improve readability and homogeneity, effect sizes (Cohen’s *d*) were calculated for all statistical tests [57]. Following Cohen [57], *d*-values ≤ .49 indicate small effects, .50 ≤ *d* ≤ .79 indicate medium effects, and *d* ≥ .80 indicate large effects. Significance level was set at *α* = 5%. All analyses were performed using SPSS version 21.0 (SPSS Inc., Chicago, IL, USA).

## Results

Mean attendance rate was high for all groups with 96% for M-SRT, 95% for M-URT, and 96% for F-URT. Seventy-five participants completed the training with eight participants (11%) dropping out. None of the drop-outs were due to intervention-related injuries. Drop-out reasons are outlined in Fig. 1. Participants reported no pain or training-related injuries. No significant baseline differences were detected (*p*s ≥ .13). Results from MMSE, CDT, GDS, and FAB-D indicated no cognitive impairments. Analysis of FQoPA revealed that participants were physically active (Table 1). Data from one participants’ gait test could not be analysed, therefore only seventy-four participants were included into gait analysis. Descriptive values of the intervention programmes for pre- and post-testing are presented in Table 3. Detailed results of the statistical analyses are outlined in Tables 4 and 5. Participants showed a reduction in their fear of falling over time (*d* = .54), but no differences between groups were detected. Results of pre- and post-assessment have can be found as Additional file 1.

**Table 3:** Descriptive values of the intervention programmes for pre- and post-testing

Variables	M-SRT (*n* = 27)				∆%	M-URT (*n* = 26)				∆%	F-URT (*n* = 22)				∆%
	pre		post			pre		post			pre		post		
	M	SD	M	SD		M	SD	M	SD		M	SD	M	SD	
Questionnaire															
Falls efficacy scale - international	20.2	3.9	20.0	4.3	−1	20.5	4.0	19.2	3.2	−6	20.1	4.4	18.5	3.0	−8
Muscle strength															
Isometric leg extension strength (N)	785	335	897	367	14	851	345	1071	359	26	867	366	995	400	15
Hand grip strength (N)	296	83	293	71	−1	318	91	316	85	−1	306	73	301	81	−1
Muscle power															
Chair rise test (s)	10.1	2.7	8.3	2.2	−18	8.5	1.9	7.8	2.0	−8	9.4	1.9	7.8	1.5	−17
Chair rise test AIREX^®^ (s)	10.9	3.0	9.0	2.5	−18	9.6	2.8	8.7	2.2	−9	10.2	2.0	8.5	1.7	−16
Stair ascent time (s)	3.9	0.8	3.6	0.7	−7	3.6	0.8	3.4	0.5	−4	3.8	0.8	3.5	0.6	−6
Stair descent time (s)	3.1	0.8	2.9	0.6	−8	3.1	0.6	2.8	0.4	−10	3.1	0.9	2.8	0.5	−10
Stair ascent power (W)	321.2	101.7	327.3	81.8	5	332.4	75.3	341.4	68.2	3	314.3	87.8	315.8	64.6	<1
Stair descent power (W)	383.2	108.1	410.2	107.1	7	386.9	89.1	421.9	88.2	9	387.8	126.1	404.1	104.9	4
Proactive balance															
Timed up and go test (s)	5.7	1.2	5.3	1.0	−8	5.2	0.9	5.0	0.5	−4	5.4	1.1	5.1	0.7	−6
Functional reach test (cm)	36.9	4.9	40.2	5.5	9	38.1	4.5	40.7	4.4	7	38.3	5.4	44.1	5.5	15
Functional reach test AIREX^®^ (cm)	36.5	5.2	39.7	5.5	9	38.5	5.2	42.2	4.7	10	37.7	6.8	44.0	5.7	17
Dynamic steady-state balance															
Stride velocity (m/s)	1.4	0.2	1.6	0.2	8	1.5	0,2	1.6	0.2	6	1.5	0.1	1.5	0.2	6
Stride velocity CV (%)	2.8	0,9	2.9	1.4	−3	2.8	1.0	2.3	1.3	22	2.6	0.9	2.6	0.9	2
Stride length (cm)	146.2	13.1	152.3	14.6	4	150.2	13.7	155.9	15.7	4	147.6	10.8	152.7	13.3	3
Stride length CV (%)	2.5	0.8	2.8	1.5	−12	2.3	0.9	2.0	1.0	17	2.2	0.6	1.9	0.7	15
Stride width (cm)	11.5	2.6	11.6	2.8	<1	12.6	3.3	12.3	3.6	−3	11.6	2.8	11.3	2.7	−3
Stride width CV (%)	22.6	9.8	24.7	10.8	9	26.2	11.0	29.3	11.3	12	24.1	9.9	23.9	9.5	−1
Double support (%)	10.4	3.6	9.2	3.6	−13	10.3	3.6	9.5	3.5	−8	10.0	3.2	9.9	3.8	−1
Double support CV (%)	11.5	6.8	13.9	13.3	−17	10.3	4.7	12.8	9.2	−20	12.2	6.5	13.9	14.1	−12
Reactive balance															
Push and release test (score) [median, IQR]	1.0	1.0	0.3	0.7	0.7	1.0	1.0	0.5	0.7	0.5	1.3	1.1	0.3	0.3	1

**Table 4:** Statistical analysis (*p*-values and effect sizes)

Variables	3 × 2 ANOVA			Pre-post analysis (post-hoc)		
	Main effect: Time	Main effect: Group	Interaction effect: Time × Group	M-SRT	M-URT	F-URT
	*p*-value (effect size *d*)			*p*-value (effect size *d*)		
Questionnaires						
Falls efficacy scale – international	.024 (.54)	.428 (.22)	.392 (.33)			
Muscle strength						
Isometric leg extension strength	<.001 (2.07)	.439 (.14)	.024 (.66)	<.001 (.30)	<.001 (.55)	<.001 (.32)
Hand grip strength	.253 (.27)	.592 (.24)	.979 (.06)			
Muscle power						
Chair rise test	<.001 (1.67)	.164 (.45)	.026 (.65)	<.001 (.74)	.004 (.34)	<.001 (.93)
Chair rise test AIREX^®^	<.001 (1.65)	.416 (.31)	.105 (.50)	<.001 (.69)	<.006 (.32)	<.001 (.95)
Stair ascent time	.017 (.58)	.428 (.31)	.835 (.14)			
Stair descent time	<.001 (.94)	.902 (.11)	.959 (.06)			
Stair ascent power	.278 (.26)	.546 (.26)	.785 (.26)			
Stair descent power	.002 (.76)	.943 (.09)	.656 (.22)			
Proactive balance						
Timed up and go test	<.001 (.89)	.211 (.42)	.460 (.29)			
Functional reach test	<.001 (1.68)	.130 (.48)	.069 (.56)	.002 (.62)	.002 (.60)	<.001 (1.03)
Functional reach test AIREX^®^	<.001 (1.65)	.123 (.31)	.071 (.51)	.002 (.61)	<.001 (.73)	<.001 (.99)
Dynamic steady-state balance						
Stride velocity	<.001 (1.40)	.992 (.20)	.810 (.03)			
Stride velocity CV	.293 (.26)	.248 (.40)	.248 (.40)			
Stride length	<.001 (1.40)	.559 (.28)	.902 (.20)			
Stride length CV	.538 (.14)	.016 (.70)	.104 (.52)			
Stride width	.267 (.28)	.394 (.32)	.661 (.20)			
Stride width CV	.008 (.66)	.864 (.01)	.440 (.06)			
Double support	.021 (.54)	.990 (.20)	.329 (.32)			
Double support CV	.081 (.41)	.781 (.16)	.957 (.06)			
Reactive balance	Friedman	K-W	K-W			
Push and release test	<.001 (1.46) Chi^2^ = 25.94	.947 (.08) Chi^2^ = .11	.503 (.26) Chi^2^ = 1.38			

*K-W* Kruskal-Wallis test; *M-SRT* machine based resistance training on stable surfaces; *M-URT* machine-based resistance training on unstable surfaces; *F-URT* free-weight resistance training on unstable surfaces; Cohen’s *d* values ≤ .49 indicate small, .50 ≤ *d* ≤ .79 medium and ≥ .80 large effects

**Table 5:** Statistical analysis (*df*, *F*-values and *t*-values)

Variables	3 × 2 ANOVA			Pre-post analysis (post-hoc)		
	Main effect: Time	Main effect: Group	Interaction effect: Time × Group	M-SRT	M-URT	F-URT
	*df & F*-value			*t*-value		
Questionnaires						
Falls efficacy scale – international	*F*(1, 72) = 5.33	*F*(2, 72) = .43	*F*(2, 72) = .95			
Muscle strength						
Isometric leg extension strength	*F*(1, 72) = 76.66	*F*(2, 72) = .83	*F*(2, 72) = 3.91	*t*(26) = −5.17	*t*(25) = −6.92	*t*(21) = −3.39
Hand grip strength	*F*(1, 72) = 1.33	*F*(2, 72) = .53	*F*(2, 72) = .02			
Muscle power						
Chair rise test	*F*(1, 72) = 51.53	*F*(2, 72) = 1.86	*F*(2, 72) = 3.84	*t*(26) = 4.71	*t*(25) = 3.16	*t*(21) = 4.48
Chair rise test AIREX^®^	*F*(1, 72) = 49.19	*F*(2, 72) = 1.00	*F*(2, 72) = 2.33	*t*(26) = 4.72	*t*(25) = 3.29	*t*(21) = 4.05
Stair ascent time	*F*(1, 72) = 5.97	*F*(2, 72) = .86	*F*(2, 72) = .18			
Stair descent time	*F*(1, 72) = 15.79	*F*(2, 72) = .10	*F*(2, 72) = .04			
Stair ascent power	*F*(1, 72) = 1.19	*F*(2, 72) = .61	*F*(2, 72) = .24			
Stair descent power	*F*(1, 72) = 10.29	*F*(2, 72) = .06	*F*(2, 72) = .42			
Proactive balance						
Timed up and go test	*F*(1, 72) = 14.34	*F*(2, 72) = 1.59	*F*(2, 72) = .76			
Functional reach test	*F*(1, 72) = 51.13	*F*(2, 72) = 2.10	*F*(2, 72) = 2.77	*t*(26) = −3.67	*t*(25) = −3.39	*t*(21) = −4.92
Functional reach test AIREX^®^	*F*(1, 72) = 64.61	*F*(2, 72) = 2.16	*F*(2, 72) = 2.74	*t*(26) = −3.51	*t*(25) = −5.34	*t*(21) = −5.13
Dynamic steady-state balance						
Stride velocity	*F*(1, 71) = 35.11	*F*(2, 71) = .08	*F*(2, 71) = .21			
Stride velocity CV	*F*(1, 71) = 1.13	*F*(2, 71) = .71	*F*(2, 71) = 1.42			
Stride length	*F*(1, 71) = 34.34	*F*(2, 71) = .59	*F*(2, 71) = .10			
Stride length CV	*F*(1, 71) = .38	*F*(2, 71) = 4.39	*F*(2, 71) = 2.34			
Stride width	*F*(1, 71) = 1.25	*F*(2, 71) = .94	*F*(1, 71) = .42			
Stride width CV	*F*(1, 71) = 7.59	*F*(2, 71) = .15	*F*(2, 71) = .83			
Double support	*F*(1, 71) = 5.56	*F*(2, 72) = .01	*F*(2, 72) = 1.13			
Double support CV	*F*(1, 71) = .38	*F*(2, 72) = .12	*F*(2, 72) = .49			

*K-W* Kruskal-Wallis test; *M-SRT* machine based resistance training on stable surfaces; *M-URT* machine-based resistance training on unstable surfaces; *F-URT* free-weight resistance training on unstable surfaces

### Training load

**Fig. 2:**
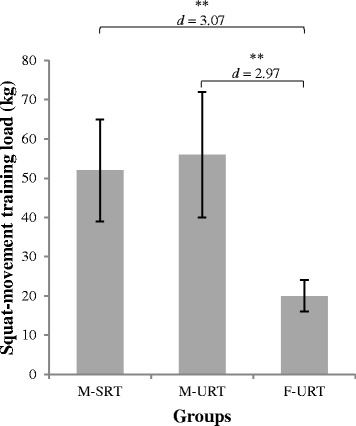
Maximal training load of the squat movement during the last training block. Note: Error bars indicate the standard deviation of the mean training load; * indicates a significant difference (*p* ≤ .05), ** indicates a highly significant difference (*p* ≤ .001); Cohen’s *d* values ≤ .49 indicate small, .50 ≤ *d* ≤ .79 medium and ≥ .80 large effects; M-SRT = machine based stable resistance training; M-URT = machine based unstable resistance training; F-URT = machine based unstable resistance training

The absolute training intensity of the squat exercise during the last week of the intervention period was assessed. M-SRT exercised with 52 ± 13 kg, M-URT with 56 ± 16 kg, and F-URT with 20 ± 16 kg, respectively. Statistically significant differences were found among groups (*F*(2, 74) = 57.02, *p* < .001, *d* = 2.3). Post-hoc analyses located the following significant differences: F-URT vs. M-SRT (*t*(47) = 10.67, *p* < .001, *d* = 3.07) and F-URT vs. M-URT (*t*(46) = 10.26, *p* < .001, *d* = 2.97) in favour of F-URT. Comparison between M-SRT and M-URT remained non-significant (*t*(51) = −.96, *p* = .344, *d* = .03) (Fig. 2).

### Muscle strength and power

**Fig. 3:**
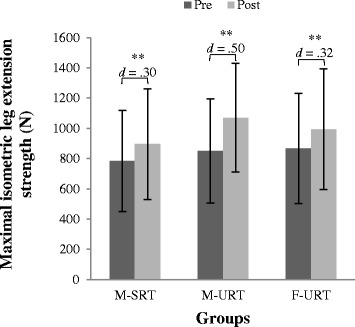
Training-related performance changes on maximal isometric leg extension strength from pre to post testing. Note: Error bars indicate the standard deviation of the mean training load; * indicates a significant difference (*p* ≤ .05), ** indicates a highly significant difference (*p* ≤ .001); Cohen’s d values ≤ .49 indicate small, .50 ≤ d ≤ .79 medium and ≥ .80 large effects; M-SRT = machine based stable resistance training; M-URT = machine based unstable resistance training; F-URT = machine based unstable resistance training

All groups showed improvements over time in proxies of lower extremity muscle strength (range Cohen’s *d*: .30–.55), with meaningfully better improvements for M-URT (Fig. 3). Hand grip strength remained non-significant in any aspect.

**Fig. 4:**
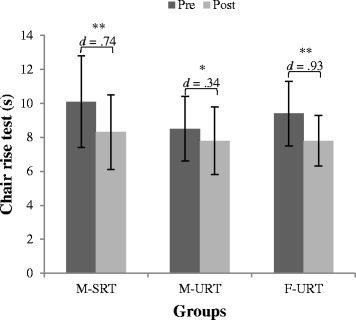
Training-related performance changes in the chair rise (stable) test from pre to post testing. Note: Error bars indicate the standard deviation of the mean training load; * indicates a significant difference (*p* ≤ .05), ** indicates a highly significant difference (*p* ≤ .001); Cohen’s d values ≤ .49 indicate small, .50 ≤ d ≤ .79 medium and ≥ .80 large effects; M-SRT = machine based stable resistance training; M-URT = machine based unstable resistance training; F-URT = machine based unstable resistance training

In terms of lower-extremity muscle power, results of the chair rise test showed improvements for all groups over time (range Cohen’s *d*: .32–.95) (Fig. 4). Though, significantly best improvements on stable surface and under the constraint of the AIREX^®^ condition were provided by F-URT. The statistical analysis revealed a significant main effect of “time” (*d* = .58) for the stair ascend task, but neither a main effect “group” nor any interaction effect could be found for stair ascend and decent.

### Balance assessment

The functional reaching distance was assessed under two conditions, stable surface and while standing on an AIREX^®^ balance pad. All groups improved reaching distance in both conditions (range Cohen’s *d*: .60–1.03), however F-URT revealed the highest effects. Although, the analysis indicated a significant main of “time” (*d* = .89) for the TUG, no interaction effect was found, indicating similar improvements across groups. The gait analysis revealed meaningful main effects of “time” (range Cohen’s *d*: .54–1.40) for stride velocity, stride length, stride width CV and double support and an main effect “group” for stride length CV (*d* = .70). No interaction effects were detected. Non-parametric analysis of the push and release test revealed meaningful improvements over time (*d* = 1.46) but little effects between groups and interaction effects.

## Discussion

This study compared the effects of lower-extremity resistance training on stable and unstable surfaces on measures of strength, power and balance in healthy older adults. The main findings of the study can be summarised as follows: (1) all training programmes represent safe (i.e., no training-related injuries occurred) and feasible training programmes for older adults, with high attendance rates (96% for M-SRT, 95% for M-URT, and 96% for F-URT) and low drop-out rates (M-SRT: 7%, M-URT: 7%, F-URT: 15%); (2) all three interventions showed improvements on measures of lower-extremity strength (ILES), power (CRT, SCPT) and balance (proactive [TUG, FRT], dynamic steady state [stride velocity, stride length, stride width CV, double support], and reactive balance [PRT]); (3) moderate instability (M-URT) induced extra effects in lower-extremity strength (ILES) compared to M-SRT and F-URT. Furthermore, higher instability (F-URT) induced extra effects in CRT compared to M-SRT and M-URT; (4) a higher instability (i.e., F-URT as compared to M-SRT and M-URT) compensates lower training load in terms of overall training-related performance gains; (5) the observed performance changes did not differ with respect to test modality (i.e., unstable vs. stable surface condition).

### Effects of resistance training on stable vs. unstable surfaces

This study demonstrated that proxies of strength, power, and balance improved in all three intervention groups. Yet, performance gains did not differ meaningfully between groups, except for ILES, where M-URT (*d* = .55) showed the largest improvements and for CRT, with F-URT (*d* = .93) showing the largest improvements. Thus, the initial assumption was partially confirmed. These findings support previous research [11, 16, 58] on the effects of URT, showing little or no training-related differences in performance outcomes comparing URT with SRT in young adults.

In previous studies, performance changes following URT in older adults have been compared to non-exercising control groups and meaningful effects have been reported. Granacher and colleagues, for example [19], conducted a study investigating the effects of a 9-week core instability strength training programme in 32 older adults (63–80 years) on measures of trunk muscle strength, spinal mobility, dynamic balance and functional mobility. They found significant main effects of “time”. The post-hoc analyses revealed a large effect for the FRT (*d* = 1.49) and a small effect for the TUG (*d* = .49) in favour of the URT group. The greater improvements for the FRT found by Granacher and colleagues [19] might be explained by the nature of the study design. Specifically, their training programme consisted of core exercises, whereas the training programme of this study was tailored to strengthen lower-extremity muscles. Hence, better adaptations of core muscle strength might be responsible for the larger effects in the FRT. Furthermore, Seo and colleagues [21] showed that a 12-weeks Swiss ball exercise programme can enhance balance performance in elderly women (> 78 years). The URT group showed a medium effect (*d* = .42) from pre to post testing in the TUG. The slightly smaller improvements found in this study are possibly because of the already fast TUG times at baseline. Furthermore, the slower TUG times found by Seo and colleagues [21] could be due to the participants’ age (>78 years). In line with these findings are the results of Chulvi-Medrano and colleagues [20]. They investigated the effects of a lower-limb strength training programme in elderly women (> 65 years) using the unstable T-Bow^®^ device on different measures of balance. Following training, URT improved from pre- to post testing in the 8-ft-up-and-go test (*d* = 3.10), which is comparable to the TUG.

There are currently no studies available that evaluated the effects of resistance training on stable versus unstable surfaces, using additional load, on measures of lower-extremity muscle strength, power and balance in older adults. This gap in scientific literature sparks the following discussion, which will review data collected in trials with young adults. Kibele and Behm [11] for example, compared the effects of a 7-week URT versus SRT programme on measures of strength, balance, and functional performance. Forty sport science students (23 ± 1 year) conducted exercises on stable or unstable surfaces. Following training, both groups showed significant small improvements (*d* = .34) with regard to ILES, but no other apparent differences. The difference in ILES between this and the present study is possibly due to varying fitness level and age of the participants. Kibele and Behm [11] included young sports students. Because of their fitness level, they have less potential for improvement than older adults. Furthermore, Kibele and Behm [11] conducted just free-weight training using unstable surfaces, whereas most meaningful effects in regard to ILES in this study were apparent in M-URT. Given the relatively higher loads due to moderate instability, consequentially higher strains were put on participants resulting in higher strength adaptations. In line with this study’s approach, Maté-Muñoz and colleagues [58] investigated the effects of a 7-week free-weight URT and SRT in young male sport students (22 ± 1 years) on measures of strength, power, and velocity. Maté-Muñoz and colleagues [58] found a “time x group” interaction effect for leg strength, with larger improvements in URT (*d* = .67) as opposed to SRT (*d* = .45). This is in line with the present study’s findings.

### Similar improvements despite different training load

Surprisingly, improvements in training-related performance gains were quite similar in most result variables. In the light of the majority of outcomes alone, the initial assumption of a slight superiority of URT is debatable. But incorporating the factor ‘training load’, F-URT seems to stand out.

Post-hoc analyses showed a medium effect in M-URT and a small one in F-URT and M-STR for measures of lower-extremity muscle strength (ILES). On the other hand, the CRT showed a large effect in F-URT and a medium effect in M-SRT, whereas M-URT showed a small effect. This result is in line with the findings of Steib and colleagues [52], reporting large strength adaptations due to strength training in measures of absolute strength but not necessarily in functional performance (i.e., CRT). Why is that? Assessment of ILES is due to its nature an isometric test without any translational movement. The sit-to-stand movement of the CRT is more similar to free-weight squats than it is to squats conducted on a Smith-machine. According to the principle of training specificity [18], effects should consequently be more apparent in tests, which are related to the training modality. As a result, F-URT showed larger improvements in the CRT performance than the machine-based training groups. In measures of proactive balance (i.e., FRT), post-hoc analyses revealed a large effect in favour of F-URT on the stable and the unstable surface condition, whereas M-SRT (and M-URT showed medium effects. Thus, the higher degree of surface instability in F-URT may have induced additional proactive balance adaptations as compared to the other groups. In other words, the balance component to URT may put higher strains on the core and the stabilising function of the lower-extremity muscles, therefore improving balance. This might explain the higher gains in proactive balance in favour of F-URT as compared to M-SRT and M-URT.

As load measures of the squat movement revealed, resistance intensity was similar for M-URT and M-SRT, yet M-URT showed superior strength improvements. Furthermore, F-URT exercised with considerably lower loads, yet improving in line with M-SRT in ILES. Provided that strength gains due to resistance training are based on intensity [59], the present study’s results need explaining. Possible reasons for this effect may be manifold. Numerous studies have documented lower muscle force [15, 60, 61] and muscle power [62] production while performing resistance training exercises under unstable surface conditions, whilst muscle activity seems to remain similar or even higher as compared to stable surface conditions [13]. As Behm and Colado [16] pointed out, URT can be split into two components: balance and load/resistance. It seems that the balance-component and the additional resistance initiate a synergetic effect within the neuromuscular system. Thus, effective stimuli for training adaptations exercising URT can be provided despite lower loads, indicating that the higher degree of instability in F-URT compensates for the lower training load resulting in comparable performance gains. Or in the case of M-URT, the same load combined with moderate instability provides superior improvements in terms of performance improvements in comparison to the other groups.

An alternative, but not a contradictory explanation has been developed by Pijnappels and colleagues [63] and Reeves and colleagues [64]. These authors tackled the issue of similar increases in strength and functional performances despite differences in load (50 vs. 80% of the 1-RM as reported by Vincent et al. [47] and 55–60% vs. 80–90% of the 1-RM as stated by Tanimoto and colleagues [65]). Pijnappels and colleagues [63] argued that load, expressed as percentage of 1-RM, may in fact not be a good predictor of optimal strain for the elderly, because the level of neuromuscular loading of many resistance training programmes easily exceeds the daily demands of older adults and overshoots the thresholder for sufficient loading to induce adaptations. In a meta-analysis on the dose-response relationship of resistance training in older adults, Steib and colleagues [52] examined the optimal loading on measures of muscle strength, endurance, power, and functional parameters related to mobility in older adults. After reviewing 22 studies, Steib and colleagues [52] came to the conclusion that higher loads (60–80% of the 1-RM) are superior to lower intensities in terms of absolute strength gains, but not necessarily for improvements in functional performance. Since the intensity in this study was 40 to 60% of the 1-RM, training effects are more applicable to power and neural adaptations than maximal and hypertrophy based strength effects.

Consequently, if the aim is to improve muscle strength and power of the lower-extremities, resistance training using unstable surfaces with moderate instability (M-URT) is recommended as compared to ‘traditional’ M-SRT. On the other hand, if training load is a limiting factor, F-URT can be recommended, because of the instability-related reduction of maximal training load.

### Role of testing surface condition

No modality related superiority of performance changes was found when tested on stable versus unstable surfaces.

CRT and FRT were performed on stable and unstable surfaces during pre- and post-testing. Due to the principle of training specificity [18], it could have been assumed that groups will show larger improvements within their respective training modality: i.e., URT groups would perform better on the unstable surface test condition and SRT group on the stable surface condition. This assumption could not be fulfilled. Since the modality “URT” showed medium and large effects when being tested on unstable surfaces and SRT improved also with a medium-sized effect, no overall superiority can be stated.

Overall, there were no meaningful differences between test modalities. This could be the case because of similar improvements in strength, power and balance performance overall and because of the effect that the degree of instability of the AIREX^®^ pad did not induce specific adaptive responses in URT groups as opposed to the SRT group.

### Functional and clinical implications

From a functional point of view, resistance training serves a specific means within a geriatric context. As mentioned before, larger improvements in terms of absolute strength gains do not necessarily result in larger functional benefits [52]. However, including unstable surfaces within a training regime seems to have functional advantages over traditional stable resistance training. With enhanced activation of trunk muscles and stabilising function of smaller and major muscles the transfer of angular momentum and power between the lower and upper extremities is facilitated [14, 19], resulting in improved postural control. This notion is mainly supported by this study’s larger improvements of the FRT after exercising on unstable surfaces in comparison to stable surfaces.

These results should encourage geriatric practices and clinical programs to consider adding F-URT as an exercise modality to their programs. Due to the instability-related reduction of load this exercise modality is potentially suitable for a variety of pathological and frail older patients, who cannot endure high loads. Based on a recent systematic review by da Labra and colleagues [66], frail older adults benefit as much as healthy older adults from resistance training in terms of strength gains. Thus, it seems likely that the frail are as susceptible to URT as the healthy older adults of this study were found to be. Further, when administered in a progressive order with gradually increasing complexity, URT proved to be a feasible and safe training program.

### Limitations

One limitation of this study that warrants discussion is the absence of a non-exercising control group. The rationale not to include a non-exercising control group was threefold. First, the effectiveness of resistance training when applied as single means, in comparison to a non-exercising control group on measures of lower-extremity muscle strength, power and balance in older adults has been frequently reported in randomised controlled studies [48, 67], reviews [49, 51] and meta-analyses [4, 52, 68]. Second, because of the proven effectiveness, it appears unethical to withhold an effective treatment from older adults. Third, any kind of gym-based training programme is tied to attention and group dynamic factors, which can lead to performance improvements. Therefore, performance gains in favour of an intervention group cannot necessarily be attributed to the training programme, as compared to a non-exercising control group. Therefore, an active instead of a passive non-exercising control group was implemented.

Another limitation is the activity and health level of this study’s participants. Because a healthy cohort was intentionally selected for this study to reduce the number of confounding factors (e.g.; health issues and pain), generalisability of these results in frail or pathological older adults is yet to be determined.

A third limitation that warrants discussion is the lack of assessor blinding during post-test assessment. This yields a potential bias. However, only one assessor was not blinded and participants were randomly allocated to the assessors and no verbal support has been given during testing, thus minimising potential bias during data collection.

## Conclusion

This study demonstrated the feasibility and effectiveness of different types of URT (i.e., M-URT and F-URT) and SRT on measures of lower-extremity muscle strength, power and balance in healthy older adults. Although F-URT was not superior in terms of overall training improvements, because of instability-related reduction of load it is eligible for training programmes and audiences bound by the necessity of lower training loads, for example in older adults.

## Additional file

Additional file 1: This file comprises a table providing data of pre- and post-measures of the study. (XLSX 43 kb)

## Abbreviations

1-RM: One repetition maximum
CDT: Clock Drawing Test
CRT: Chair Rise Test
CV: Coefficient of variation
D: Vertical distance
*d*: Cohen’s d
FAB-D: Frontal Assessment Battery
FES-I: Falls Efficacy Scale – International
FQoPA: Freiburg Questionnaire of Physical Activity
FRT: Functional Reach Test
F-URT: Free-weight resistance training conducted on unstable surfaces
GDS: Geriatric Depression Scale
ICC: Interclass Correlation Coefficient
ILSE: Maximal isometric leg extension strength
IQR: Interquartile Range
K-W: Kruskal-Wallis
M: Body mass
MMSE: Mini-Mental-State-Examination
M-SRT: Machine-based resistance training conducted on stable surfaces
M-URT: Machine-based resistance training conducted on unstable surfaces
P: Power (watt)
PRT: Push and Release Test
*r*: Correlation coefficient
SCPT: Stair climb power test
SRT: Resistance training conducted on stable surfaces
t: Time
TUG: Timed Up and Go test
URT: Resistance training conducted on unstable surfaces
